# Prevalence of virulence factors in enterotoxigenic *Escherichia coli* isolated from pigs with post-weaning diarrhoea in Europe

**DOI:** 10.1186/s40813-016-0039-9

**Published:** 2016-09-01

**Authors:** Andrea Luppi, Mariavittoria Gibellini, Thomas Gin, Frédéric Vangroenweghe, Virginie Vandenbroucke, Rolf Bauerfeind, Paolo Bonilauri, Geoffrey Labarque, Álvaro Hidalgo

**Affiliations:** 1 Istituto Zooprofilattico Sperimentale della Lombardia e dell’Emilia Romagna (IZSLER), via Pitagora 2, Reggio Emilia, Italy; 2 Elanco Animal Health, Divisione veterinaria Eli Lilly Italia Spa, Via A. Gramsci, 731, 50019 Sesto Fiorentino, FI Italy; 3 Elanco Animal Health, 24 Boulevard Vital Bouhot, 92521 Neuilly-sur-Seine, France; 4 Elanco Animal Health Benelux, Plantijn en Moretuslei 1 -3rd floor, 2018 Antwerpen, Belgium; 5 Dierengezondheidszorg (DGZ) Vlaanderen, Industrielaan 29, 8820 Torhout, Belgium; 6 Institute of Hygiene and Infectious Diseases of Animals, Justus Liebig University, Frankfurter Str. 85-89, 35392 Giessen, Germany; 7 Elanco Animal Health, Lilly House, Priestley Road, Basingstoke, RG249NL UK

**Keywords:** *Escherichia coli*, Prevalence, ETEC, Post-weaning diarrhoea

## Abstract

**Background:**

Post-weaning diarrhoea (PWD), due to *Escherichia coli*, is an important cause of economic losses to the pig industry primarily as a result of mortality and worsened productive performance. In spite of its relevance, recent data about the prevalence of virulence genes and pathotypes among *E. coli* isolates recovered from cases of PWD in Europe are scarce.

**Results:**

This study investigates the prevalence of fimbrial and toxin genes of *E. coli* by PCR among 280 farms with PWD across Europe. A total of 873 samples collected within the first 48 h after the onset of PWD (occurring 7–21 days post weaning) were submitted to the laboratory for diagnostic purposes. Isolation and identification of *E. coli* were performed following standard bacteriological methods and PCR assays for the detection of genes encoding for fimbriae (F4, F5, F6, F18 and F41) and toxins (LT, STa, STb and Stx2e). The prevalence of fimbriae and toxins among *E. coli* isolates from cases of PWD was: F4 (45.1 %), F18 (33.9 %), F5 (0.6 %), F6 (0.6 %), F41 (0.3 %), STb (59.1 %), STa (38.1 %), LT (31.9 %) and Stx2e (9.7 %). *E. coli* isolates carrying both fimbrial and toxin genes were detected in 52.5 % of the cases (178 out of 339 isolates), with 94.9 % of them being classified as enterotoxigenic *E. coli* (ETEC). The most common virotype detected was F4, STb, LT.

**Conclusions:**

This study confirms that ETEC is frequently isolated in pig farms with PWD across Europe, with F4- and F18-ETEC variants involved in 36.1 % and 18.2 % of the outbreaks, respectively.

## Background

Post-weaning diarrhoea (PWD) in piglets remains a major cause of economic losses for the pig industry due to mortality, morbidity, decreased growth rate and cost of medication [1]. Enterotoxigenic *Escherichia coli* (ETEC) is regarded the most important cause of PWD, a pathotype characterized by the presence of fimbrial adhesins, which mediate attachment to porcine enterocytes, and enterotoxins, which disrupt fluid homeostasis in the small intestine. As a result of ETEC infection in PWD cases, mild to severe diarrhoea develops, typically within a few days after weaning, associated with dehydration, loss of body condition and increased mortality [2].

The adhesive fimbriae most commonly found in ETEC from pigs suffering PWD are those of types F4 (previously known as K88) and F18 (F107, 2134P, 8813). In addition, other adhesive fimbriae have been identified in *E. coli* isolates recovered from PWD cases, such as F5 (K99), F6 (987P), and F41, although more rarely [2–6]. The main enterotoxins detected in porcine ETEC are heat-labile toxin (LT), heat-stable toxin a (STa) and heat-stable toxin b (STb). Some strains can produce both enterotoxins and a Shiga toxin which is usually Stx2e subtype. Some authors classify these strains as ETEC rather than Shiga toxin producing *E. coli* (STEC) [2].

In spite of its relevance, only a few recent studies investigating the occurrence and the distribution of fimbriae and virulence factors among *E. coli* isolates from cases of PWD in Europe are available and in many cases they do not differentiate between *E. coli* isolates recovered from PWD cases and oedema disease or pre-weaning diarrhoea [4, 7]. Reports from Poland, Slovakia and Denmark suggest differences between countries among the prevalence of the main fimbrial types in *E. coli* isolates recovered from pigs with PWD [4, 6, 8].

A simple and easy method to determine the presence of fimbrial adhesins of ETEC is slide agglutination. This method has been particularly useful for the identification of fimbrial adhesin F4, although is less reliable for other fimbria such as F6, F5 or F41 due to their variable expression *in vitro* [2, 9]. Nowadays, genotypic analysis is commonly used to investigate the *E. coli* virotype involved in an infection and PCR genetic characterization is becoming increasingly available in veterinary diagnostic laboratories. Multiplex PCR for the detection of genes encoding for toxins (STa, STb, LT, and Stx2e) and fimbriae (F4, F5, F6, F18 and F41) is currently accessible to pig practitioners in Europe and can be an option for routine diagnostics of *E. coli*.

This study reports on the prevalence of virulence genes in enterotoxigenic *E. coli* isolates recovered from recent cases of PWD in pig farms across several countries in western and southern Europe.

## Methods

### Sampling of pig farms with cases of PWD

A total of 280 pig farms with acute cases of PWD were sampled as part of a diagnostic exercise between January 2012 and December 2014. Farms were located in the Flemish Region of Belgium and The Netherlands (*n* = 88), France (*n* = 91), Italy (*n* = 84) and Germany (*n* = 17). Sampled herds had a history of PWD occurring from 1 to 3 weeks after weaning, as evidenced by typical clinical signs: diarrhoea, decreased feed consumption, dehydration, depression and increased mortality.

Diagnostic specimens were obtained from 873 three- to five-week old pigs with diarrhoea (one sample per animal), within the first 48 h after the symptoms of PWD had occurred and before specific antimicrobial treatment had been administered. The use of zinc oxide in these outbreaks was not monitored. A detailed description of number and type of samples by country is presented in Table 1.

**Table 1:** Number of farms, samples and sample types investigated in this study by country of origin

Country	Farms n	Samples	
		n	Type^a^
Belgium and The Netherlands	88	160	S, P
France	91	455	S
Germany	17	99	S
Italy	84	159	S, F, I, P
Total	280	873	

^a^ Sample type: S, rectal swabs; F, faeces; I, intestinal content; P, entire piglets

### Isolation and characterization of Escherichia coli isolates

Specimens were processed using standard procedures for isolation and characterization of intestinal *E. coli* [10]. Briefly, samples were plated on selective media and on tryptose soy agar (TSA) medium supplemented with 5 % of defibrinated ovine blood and incubated aerobically overnight at 37 °C. Haemolytic activity was evaluated and single coliform colonies were further characterized by biochemical methods.

### DNA extraction and characterization of virulence factors by PCR

DNA samples were prepared from one up to five haemolytic and/or not haemolytic *E. coli* colonies and used to perform a multiplex PCR for the detection of fimbrial and toxin genes, including those encoding for F4 (K88), F5 (K99), F6 (987P), F18, F41, LT, STa, STb and Stx2e, but not discriminating between F4ab, F4ac and F4ad. The methodology used for the identification of these virulence genes has been described previously [11].

When *E. coli* isolates with the same combination of virulence factors (virotype) were detected in the same herd they were considered duplicates of an *E. coli* prototype strain. The prototype but not the duplicates were considered for prevalence calculations. *E.coli* strains carrying both fimbrial and toxin genes were classified into pathotypes. Isolates encoding at least one of the investigated enterotoxins together with Stx2e and F18 fimbriae were classified as ETEC based on the PWD clinical signs observed, as previously proposed [2]. Isolates carrying genes for adhesive fimbriae and Stx2e were classified as STEC.

## Results

A total of 844 DNA samples belonging to the same number of *E. coli* isolates and representing all of the 280 PWD-affected pig farms included in this study were analysed by PCR. These isolates were classified as being positive or negative for the presence of one or more virulence genes (F4, F5, F6, F18, F41, LT, STa, STb and Stx2e). Following these criteria and excluding isolates which were considered duplicates within herds, the prevalence of virulence genes was calculated. Eighty nine per cent (302 out of 339) of *E. coli* isolates carried at least one virulence gene, while 10.9 % (37 out of 339) resulted negative for all of the virulence genes investigated. Fimbrial genes were identified in 75.8 % (257 out of 339) and toxin genes in 65.8 % (223 out of 339) of the isolates. In 28.3 % (96 out of 339) of the *E. coli* isolates, a single virulence gene was detected.

The prevalence of fimbrial and toxin genes in non-duplicate *E. coli* isolates from PWD-affected pig farms across Europe is shown in Table 2. Overall, the adhesive fimbriae most commonly detected was F4 (45.1 %), followed by F18 (33.9 %). Sixteen isolates possessed genes for two types of fimbriae. The combination of F4 and F18 was detected in 15 isolates, with and without toxin genes, whereas F5 and F41 in one isolate. The most prevalent toxin was STb (59.1 %), followed by STa (38.1 %) and LT (31.9 %).

**Table 2:** Prevalence of examined genes for fimbriae and toxins among 339 isolates of *E. coli* recovered from pigs with PWD across Europe

Country and number of isolates tested	Percentage (number) of positive *E. coli* isolates^a^								
	Fimbriae					Toxins			
	F4	F5	F6	F18	F41	LT	STa	STb	Stx2e
Belgium and The Netherlands (*n* = 100)	51.0 (51)	1.0 (1)	1.0 (1)	42.0 (42)	-	14.0 (14)	22.0 (22)	30.0 (30)	5.0 (5)
France (*n* = 91)	47.3 (43)	-	-	35.2 (32)	-	45.1 (41)	40.7 (37)	76.9 (70)	19.8 (18)
Germany (*n* = 64)	14.1 (9)	-	-	14.1 (9)	-	9.4 (6)	26.6 (17)	57.8 (37)	3.1 (2)
Italy (*n* = 84)	59.3 (50)	1.2 (1)	1.2 (1)	38.1 (32)	1.2 (1)	56.0 (47)	63.1 (53)	71.4 (60)	9.5 (8)
All countries (*n* = 339)	45.1 (153)	0.6 (2)	0.6 (2)	33.9 (115)	0.3 (1)	31.9 (108)	38.1 (129)	59.1 (197)	9.7 (33)

^a^ Isolates carrying genes for two fimbriae or two or more toxins were identified in 16 and 178 cases respectively

Based on the presence of genes for both fimbriae and toxins, 52.5 % (178 out of 339) of the *E. coli* isolates were classified into pathotypes (Table 3). Within these, ETEC was the most frequent pathotype with 94.9 % of the isolates (169 out of 178). The ETEC virotypes most commonly detected were (i) F4, STb, LT; (ii) F4, STa, STb, LT and (iii) F4, STa, STb, found in 49, 28 and 18 isolates, respectively. Adhesive fimbria F18 was mainly detected in combination with genes encoding for (i) STa, STb and (ii) STa, STb, Stx2e. Among the ETEC, 23 isolates encoded for Stx2e in addition to enterotoxins and fimbriae. Nine isolates were classified as STEC, harbouring only genes for F18 and Stx2e.

**Table 3:** Distribution of encoded virulence factor combinations among 178 *E. coli* isolates from European cases of PWD classified as ETEC or STEC in decreasing order of prevalence

Virulence factor combination	Number of positive *E. coli* isolates					
	Belgium and The Netherlands	France	Italy	Germany	All countries	
	n	n	n	n	n	%
F4, STb, LT	7	21	17	4	49	27.5
F4, STa, STb, LT	3	10	15	-	28	15.7
F4, STa, STb	9	2	3	4	18	10.1
F18, STa, STb	7	6	3	-	16	9.0
F18, STa, STb, Stx2e	1	6	8	1	16	9.0
F18, Stx2e	4	4	-	1	9	5.1
F18, STa, STb, LT	-	-	7	-	7	3.9
F18, STa	1	-	5	-	6	3.4
F18, STb, LT	1	2	-	-	3	1.7
F4, F18, STa, STb, LT	-	1	2	-	3	1.7
F4, F18, STa, STb, Stx2e	-	3	-	-	3	1.7
F4, LT	3	-	-	-	3	1.7
F18, STa, LT	-	-	2	-	2	1.1
F4, F18, STa, STb	-	2	-	-	2	1.1
F4, STa	-	-	2	-	2	1.1
F4, STb	-	-	2	-	2	1.1
F18, STb	-	1	-	-	1	0.6
F18, STb, LT, Stx2e	-	1	-	-	1	0.6
F4, F18, STa	-	-	1	-	1	0.6
F4, F18, STa, LT	-	-	1	-	1	0.6
F4, F18, STb, LT	-	1	-	-	1	0.6
F4, F18, STb, LT, Stx2e	-	1	-	-	1	0.6
F4, F18, STa, STb, LT, Stx2e	-	1	-	-	1	0.6
F4, STa, STb, LT, Stx2e	-	1	-	-	1	0.6
F5, STb	1	-	-	-	1	0.6

*E.coli* characterized as ETEC were haemolytic in 97.6 % of the cases. The remaining 2.4 % non-haemolytic ETEC isolates, for which haemolytic activity was consistently tested, were recovered in France, Italy and Germany, sharing the same virotype: F4, STa, STb. All STEC isolates (F18, Stx2e) were haemolytic.

ETEC was found in 59.6 % of the 280 PWD-affected farms investigated across Europe. F4-ETEC was the main fimbrial type, being identified as single virotype in 36.1 % of the farms. F18-ETEC was isolated in 18.2 % of the farms. In 4.6 % of the farms, an enterotoxigenic *E. coli* virotype harbouring both F4 and F18 fimbriae was found. Two different fimbrial subtypes of ETEC were identified in one farm, F4-ETEC (F4, STa, STb, LT) and F18-ETEC (F18, STa, STb). An itemization of ETEC prevalence among farms with PWD by country is shown in Table 4.

**Table 4:** Prevalence of ETEC fimbrial subtypes among 280 pig farms with cases of PWD examined in Europe

ETEC subtype	Percentage^a^ (number) of affected pig farms in a given country				
	Belgium and The Netherlands	France	Italy	Germany	All countries^b^
F4-ETEC	23.9 (21)	37.4 (34)	46.4 (39)	41.2 (7)	36.1 (101)
F18-ETEC	10.2 (9)	17.6 (16)	29.8 (25)	5.9 (1)	18.2 (51)
F4,F18-ETEC	-	9.9 (9)	4.8 (4)	-	4.6 (13)
F5-ETEC	1.1 (1)	-	-	-	0.4 (1)
F4-ETEC & F18-ETEC	1.1 (1)	-	-	-	0.4 (1)
All subtypes	36.4 (32)	64.8 (59)	81.0 (68)	47.1 (8)	59.6 (167)

^a^ Percentage of positive farms over total farms investigated in a given country: Belgium and The Netherlands, 88; France, 91; Italy, 84 and Germany, 17

^b^ Percentage of positive farms over all farms investigated (*n* = 280)

## Discussion

The present study describes the prevalence of fimbrial and toxin genes detected in *E. coli* isolated from piglets in the early stages of PWD across Belgium, The Netherlands, France, Germany and Italy. From the different adhesin fimbriae investigated, F4 was the type most commonly detected, followed by F18 (45.1 % and 33.9 % of the isolates, respectively). Similarly, a higher prevalence of F4 than F18 fimbriae has been reported before in other European countries including Denmark [4], Slovakia [12] and Czech Republic [13]. Nevertheless, data from Slovakia and Poland have shown a higher prevalence of F18 fimbriae [6, 14]. Outside Europe, a higher prevalence of F18-ETEC has been reported in Cuban pigs with diarrhoea [15], while F4 was the most common fimbriae associated to the prevalent *E.coli* serogroups (O149 and O141) causing diarrhoea in Australian pigs [16]. The F4 fimbrial gene was reported in 64.6 % of *E.coli* strains isolated from pigs with diarrhoea in the United States [17].

A small number of isolates containing F5, F6 and F41 genes were also found in our study. Although these adhesive fimbriae are usually found in *E. coli* isolates recovered from younger pigs, its detection in isolates obtained from post-weaning pigs has been described previously [3–6]. Regarding enterotoxins, the most prevalent one was STb (59.1 % of isolates), usually associated to a severe fluid loss in the small intestine of weaned piglets. The prevalence of STb is in accordance with previous studies [4, 17].

In 52.5 % of the isolates, a combination of fimbriae and toxins was detected and isolates were classified accordingly into recognized pathotypes [2]. Two pathotypes, ETEC and STEC, were found among *E. coli* isolates from cases of PWD in Europe, with the vast majority being classified as ETEC (94.9 %). The high recovery rate in our study is possibly influenced by the sampling being conducted in the first 48 h after the onset of PWD and before any antibiotic treatment and thus increasing the likelihood of detecting the causal agent rather than other resident *E. coli*.

In a small number of isolates F4 and F18 fimbrial genes were detected concurrently (F4, F18-ETEC). Whereas less frequent than other subtypes, ETEC isolates encoding for more than one of the examined adhesive fimbriae have been described before [3–5, 12]. ETEC strains possessing multiple adhesins could have a pathogenetic advantage, as supposed previously [18].

In the present study the fimbrial F4 gene was strongly associated with LT, STb and with LT, STa, STb enterotoxin gene combinations (49 and 28 isolates, respectively), which is in line with a previous report from Denmark [4]. Similar results have been described concerning the association of F4 with LT and STb, but not with STa in the United States and Slovakia [6, 17]. Interestingly, a drastic increase in the prevalence of STa has been reported in Canada when F4-ETEC isolates from 1998–2001 were compared with 1974–1987 isolates [19]. Whereas our data does not allow us to make any inference about the recent emergence of an F4-ETEC virotype containing the STa gene in Europe, it does suggest geographical differences among the major virotypes of F4-ETEC detected in cases of PWD. In addition, further genetic characterization is required to determine if the high prevalence of some virotypes identified in this study is the result of clonal expansion or horizontal gene transfer.

The third most frequent combination of fimbrial and toxin genes was F4, STa, STb, with 18 isolates. It is noteworthy that four non-haemolytic ETEC isolates detected in our study belonged to this virotype. These isolates originated from farms geographically dispersed (France, Italy and Germany), indicating that non-haemolytic ETEC can play a role in some of the PWD outbreaks in agreement with previous findings [4, 17]. The haemolytic phenotype is frequently used as an indication of *E. coli* pathogenicity among isolates recovered from PWD cases. However, non-haemolytic isolates, even if uncommon, should not be discarded in the diagnostic process particularly when clinical and pathological findings are indicative of PWD.

Twenty eight per cent of the isolates encoded for a single virulence factor and 10.9 % lacked any of the virulence genes investigated, being considered non-pathogenic. Isolates carrying at least a toxin without any known fimbriae and vice versa have been already described [6, 20]. The possible role of these strains in the development of PWD may require further investigation in order to evaluate the presence of other virulence factors such as adhesins or enterotoxins that may be involved in the pathogenesis of the disease. For example, AIDA (adhesin involved in diffuse adherence) has been associated with *E. coli* strains recovered from piglets with diarrhoea, and there is evidence that it is causatively involved in diarrhoea experimentally induced in colostrum-deprived newborn piglets with STb encoding *E. coli* [21]. However, evidence for any effect in elder or conventionally reared piglets is still lacking. Similarly, the toxin EAST1 (enteroaggregative *E. coli* heat stable enterotoxin) is commonly detected among porcine ETEC although its role in PWD remains yet to be clarified [13, 22]. Likewise, we cannot exclude the presence of enteropathogenic *E. coli* (EPEC) among those isolates or the involvement of other pathogens in the cases of PWD described in this study. EPEC can also cause PWD in pigs although it does not possess any of the classic virulence factors of ETEC [2].

In this study, 167 of the farms investigated (59.6 %) were positive for the presence of ETEC in samples from pigs with diarrhoea. Moreover, the examination of ETEC fimbrial subtypes revealed that F4-ETEC is the main subtype associated with PWD cases in Europe, followed by F18-ETEC. A higher prevalence of PWD-affected farms with F4-ETEC compared to F18-ETEC was consistently shown in all countries included in this study, with approximately twice as many outbreaks being associated with F4-ETEC as with F18-ETEC. In spite of its clinical relevance, the prevalence of pathotypes and subtypes associated with PWD outbreaks is not usually reported as a proportion of clinical cases at farm level, but as percentage of isolates investigated [3, 8, 12, 13].

## Conclusions

Our study confirms that ETEC is the main pathotype involved in clinical cases of PWD in European pig farms and that F4-ETEC is the main fimbrial subtype, followed by F18-ETEC.

The information presented in this study has practical relevance for the control of PWD in European pig producing systems. A correct sampling of diseased pigs, laboratory confirmation of the aetiological agent and associated virulence factors are required in order to achieve an early diagnosis and understand the role of *E. coli* at the onset of PWD outbreaks.

## Abbreviations

AIDA, adhesin involved in diffuse adherence; EAST1, enteroaggregative *E. coli* heat stable enterotoxin; EPEC, enteropathogenic *E.coli*; ETEC, enterotoxigenic *E.coli*; LT, heat-labile toxin; PWD, post-weaning diarrhoea; STa, heat-stable toxin a; STb, heat-stable toxin b; STEC, Shiga toxin-producing *E. coli*; Stx2e, Shiga toxin subtype 2e; TSA, tryptose soy agar
